# Azilsartan Ameliorates Diabetic Kidney Disease Through Modulation of Inflammation, Pyroptosis, and Mitochondrial Dysfunction

**DOI:** 10.1155/jdr/2555220

**Published:** 2026-09-09

**Authors:** Wang Li, Fang Hao, Xu Lijia

**Affiliations:** ^1^ Department of Emergency Medicine, The Second Affiliated Hospital of Zhejiang University School of Medicine, Hangzhou, China, z2hospital.com; ^2^ Intensive Rehabilitation Department, Linping District Hospital of Traditional Chinese Medicine, Hangzhou, Zhejiang, China; ^3^ Department of Respiratory Medicine, Linping District Hospital of Traditional Chinese Medicine, Hangzhou, Zhejiang, China

**Keywords:** Azilsartan, diabetic nephropathy, mesangial cells, mitochondrial reactive oxygen species, pyroptosis

## Abstract

**Objective:**

To investigate the protective effects of Azilsartan (AZL) on kidney injury in diabetic nephropathy (DKD) mice and high glucose (HG)‐induced mesangial cell damage, and to clarify whether these effects are mediated through the regulation of mitochondrial reactive oxygen species (mtROS) and NLRP3 inflammasome‐mediated pyroptosis.

**Methods:**

SPF male C57BL/6 mice, aged 6–8 weeks, were adapted for 1 week and randomly divided into a control group and a DKD group. The DKD model was established through right nephrectomy combined with a high‐fat diet and intraperitoneal injection of streptozotocin (STZ, 50 mg/kg). After successful modeling, the two groups were further divided into a saline subgroup and an AZL intervention subgroup, with continuous intervention for 8 weeks. In vitro, mesangial cells from mice during the logarithmic growth phase were divided into normal glucose (NG) and HG groups, further setting up saline control and AZL intervention subgroups. MitoQ, a mitochondrial‐targeted antioxidant, was used to verify the role of mtROS. Parameters including systolic blood pressure, blood glucose, serum creatinine, and urinary albumin‐to‐creatinine ratio (UACR) were measured in mice. Kidney pathology was observed by PAS staining. Western blotting was used to detect the expression of NLRP3, cleaved‐Caspase1, GSDMD‐N, and other pyroptosis‐related proteins in kidney tissues and mesangial cells. ELISA was used to measure the concentration of IL‐1*β* in serum and cell supernatants. Mitochondrial morphology, membrane potential, and mtROS levels in mesangial cells were observed using laser confocal microscopy. ATP content in cells was measured by chemiluminescence, and cell injury was assessed by LDH assay.

**Results:**

In vivo, compared to the control group, the DKD+Saline group showed significantly increased systolic blood pressure, blood glucose, serum creatinine, and UACR, with evident mesangial matrix expansion and enhanced activation of the NLRP3 inflammasome and pyroptosis. AZL intervention significantly improved these abnormalities. In vitro, HG induced mitochondrial structural damage, decreased membrane potential, reduced ATP generation, and increased mtROS levels in mesangial cells while also activating NLRP3 inflammasome‐mediated pyroptosis. AZL intervention significantly reversed these changes, and MitoQ, by inhibiting mtROS, mimicked the protective effects of AZL.

**Conclusion:**

AZL improves renal function and pathology in DKD mice, potentially through both hemodynamic (blood pressure lowering) and nonhemodynamic mechanisms. In vitro evidence indicates that AZL suppresses HG‐induced mesangial cell pyroptosis by inhibiting mtROS generation and NLRP3 inflammasome activation.

## 1. Introduction

Diabetic nephropathy (DKD) is one of the most severe and common complications of diabetes, characterized by progressive decline in kidney function and structural damage. It is the leading cause of end‐stage renal disease globally and significantly increases morbidity and mortality among diabetic patients [1]. The pathophysiological mechanisms involve complex interactions of metabolic disturbances, inflammation, and fibrosis, with hyperglycemia as the primary risk factor. Inflammation activation and abnormal cellular stress pathways are central to disease progression, leading to endothelial dysfunction and tissue fibrosis, ultimately resulting in renal failure [2–4]. Pyroptosis, a form of inflammation‐driven programmed cell death, plays a critical role in the pathogenesis of DKD [5]. Its hallmark features include inflammasome activation leading to cell membrane perforation, cell rupture, and the release of proinflammatory factors such as IL‐1*β*. The activation of the NLRP3 inflammasome and its downstream signaling components, Caspase‐1 and GSDMD, are core events that exacerbate local kidney inflammation and accelerate kidney injury [6]. Azilsartan (AZL), a highly selective angiotensin II Type 1 receptor antagonist, can mitigate angiotensin II‐mediated kidney damage. However, its anti‐inflammatory and antifibrotic mechanisms in kidney protection remain unclear [7]. Mitochondrial dysfunction and excessive generation of mitochondrial reactive oxygen species (mtROS) are crucial in the development of DKD, and targeting the regulation of mtROS represents a potential strategy to delay the progression of DKD [8]. This study is aimed at investigating the effects of AZL on inflammatory mediators, pyroptosis markers, and mtROS in a DKD mice model.

## 2. Materials and Methods

### 2.1. Experimental Animals

SPF‐grade male C57BL/6 mice, aged 6–8 weeks, weighing 20–22 g, were purchased from the Shanghai Laboratory Animal Center, Chinese Academy of Sciences (License No.: SCXK 2013‐0016). The mice were housed in standard SPF‐grade animal rooms with a temperature of 22°C ± 2°C, humidity of 50% ± 10%, and a 12‐h light/dark cycle. They were allowed free access to food and water and underwent a 1‐week acclimatization period before the experiments.

### 2.2. Experimental Cells

Mice glomerular mesangial cells (MMCs) were purchased from the Cell Bank of the Chinese Academy of Sciences. The cells were cultured in DMEM high‐glucose (HG) medium containing 10% fetal bovine serum, 100 U/mL penicillin, and 100 *μ*g/mL streptomycin in a 37°C 5% CO_2_ incubator with saturated humidity. Cells in the logarithmic growth phase were used for subsequent experiments.

### 2.3. Reagents and Consumables

AZL (purity ≥ 98%, Y10788) was purchased from Shanghai YuanYe Biotechnology Co. Ltd. Streptozotocin (STZ, purity ≥ 98%, S0130) was purchased from Sigma–Aldrich, United States, and was freshly prepared using citrate buffer (pH 4.5). Mitoquinone (MitoQ), a mitochondrial‐targeted antioxidant (purity ≥ 99%, ab146248), was purchased from Abcam, United Kingdom. HG DMEM (G4511‐500ML) and normal‐glucose (NG) DMEM (G4520‐500ML) were purchased from Wuhan Saiweier Biotechnology Co. Ltd. Fetal bovine serum (S9020) was purchased from Beijing Solarbio Technology Co. Ltd. Penicillin–streptomycin double‐antibody solution (AC‐1001039) was purchased from Jiangsu Azus Biotechnology Co. Ltd. RIPA lysis buffer (with protease inhibitor and phosphatase inhibitor, P0013B), bicinchoninic acid (BCA) protein quantification kit (P0010), SDS‐PAGE gel preparation kit (P0012A), polyvinylidene fluoride (PVDF) membrane (IPVH00010), and enhanced chemiluminescence (ECL) detection solution (P0018FS) were purchased from Shanghai Biotian Biotechnology Co. Ltd. Primary antibodies against NLRP3 (15101S), pro‐Caspase1 (24232S), cleaved‐Caspase1 (89332S), GSDMD‐FL (10937S), GSDMD‐N (13878S), *β*‐actin (4970S) and secondary antibodies HRP‐conjugated goat antirabbit IgG (7074S), HRP‐conjugated goat anti‐mice IgG (7076S) were purchased from Cell Signaling Technology (CST), United States. The cleaved‐Caspase1 (sc‐398715), PDGFR*β* (sc‐374573) and NLRP3 (sc‐518122) for immunostaining were purchased from Santa Cruz Biotechnology. MitoTracker Red mitochondrial dye (M7512), JC‐1 mitochondrial membrane potential detection kit (T3168), mitoSOX Red mtROS detection probe (M36008), Hoechst 33342 nuclear dye (H3570), and ATP assay kit (A22066) were purchased from Invitrogen, United States. IL‐1*β* ELISA kit (ml106733) and LDH cytotoxicity detection kit (ml‐E12839) were purchased from Shanghai Meilian Biological Technology Co. Ltd. Periodic acid–Schiff (PAS) staining kit (G1281) and 4% paraformaldehyde fixative (P1110) were purchased from Beijing Solarbio Technology Co. Ltd.. TRIzol was purchased from Invitrogen, United States. PrimeScript II 1st Strand cDNA Synthesis Kit was purchased from Takara, Japan. Serum creatinine assay kit (C011‐2‐1) was purchased from Nanjing Jiancheng Bioengineering Institute. Citrate buffer (pH 4.5, C1011), TBST wash buffer (P0023B), and 5% skimmed milk (D8340) were analytical grade and purchased from China National Pharmaceutical Group Chemical Reagents Co. Ltd. Gel imaging system (ChemiDoc XRS+) was purchased from Bio‐Rad, United States. Automatic biochemical analyzer (7600‐020) was purchased from Hitachi, Japan. Fluorescence microscope (BX53) was purchased from Olympus, Japan. Transmission electron microscope (JEM‐1400Plus) was purchased from JEOL, Japan.

### 2.4. Experimental Animal Grouping and Treatment

Male C57BL/6 mice, aged 6–8 weeks, were acclimatized for 1 week and then randomly divided into the control (Ctrl) group and the DKD group. The Ctrl group was fed a regular chow diet and no modeling treatment was applied. The DKD group underwent right nephrectomy, followed by a 1‐week recovery period. Then, the mice were intraperitoneally injected 50 mg/kg STZ for three times and fed a high‐fat diet (HFD). Two weeks after STZ conduct, the random blood glucose level was measured. Moreover, mice with blood glucose levels higher than 16.7 mmol/L were considered to have developed diabetes.

After successful modeling, the Ctrl and DKD groups were further subdivided into saline and AZL groups (*n* = 5 per group). Starting from the 4th week after STZ injection, the groups were continuously gavaged until the 12th week: Ctrl+Saline and DKD+Saline groups received equal volumes of saline, whereas Ctrl+AZL and DKD+AZL groups received AZL (10 mg/kg/d) by gavage.

At the end of the treatment period, all mice were euthanized, and 24‐h urine samples were collected before euthanasia. Blood was collected from the orbital sinus to prepare serum. After euthanasia, kidneys were rapidly dissected, with part of the tissue fixed in 4% paraformaldehyde for subsequent morphological analysis, and the remaining tissue was stored at −80°C for further protein analysis.

### 2.5. Experimental Cell Grouping and Treatment

Logarithmic phase MMCs were seeded into appropriate culture plates and cultured until cell confluence reached 70%–80%. The medium was then replaced with serum‐free medium for synchronization for 12 h. Subsequently, the cells were divided into the NG group and the HG group, and further divided into saline and AZL subgroups, resulting in 4 experimental groups: NG+Saline, NG+AZL, HG+Saline, and HG+AZL.

In the NG group, cells were cultured in DMEM medium containing 5.5 mmol/L glucose, whereas in the HG group, cells were cultured in DMEM medium containing 30 mmol/L glucose to establish the HG injury model. In the AZL group, AZL was added to a final concentration of 10 *μ*mol/L, whereas the saline group received an equal volume of saline. To determine whether AZL protects mesangial cell injury by inhibiting mtROS, MitoQ (25 *μ*M) was added to the culture containing HG, with or without AZL. All groups were incubated at 37°C, 5% CO_2_ for 48 h, with daily observation of cell morphology changes. After the incubation period, cells and supernatants were collected for mitochondrial function‐related assays and analysis.

### 2.6. Observation Indicators

#### 2.6.1. General and Biochemical Indicators in Mice

During the experiment, starting from the 0th week after STZ injection, systolic blood pressure (SBP) was measured weekly using the tail‐cuff method, and fasting blood glucose levels were monitored from the tail vein to dynamically track the changes in blood pressure and glucose levels in each group. At the endpoint of the experiment (12 weeks post‐STZ injection), blood and 24‐h urine samples were collected. Serum creatinine concentration was measured using an enzyme colorimetric method, and urine albumin concentration was detected using ELISA. The urine albumin‐to‐creatinine ratio (UACR) was calculated by combining the levels of urinary albumin and creatinine to assess glomerular filtration function and early kidney damage.

#### 2.6.2. Renal Tissue Morphology

At the experiment endpoint, mice kidney tissues were collected, fixed in 4% paraformaldehyde, and embedded in paraffin to make sections. PAS staining was performed, and pathological changes, such as mesangial matrix expansion in the glomerulus, were observed under a light microscope. Image analysis was conducted using ImageJ software, and the percentage of glomerular mesangial matrix expansion was quantitatively calculated.

#### 2.6.3. Detection of Relevant Protein Expression in Renal Tissue

Western blotting was used to detect the expression levels of relevant proteins (NLRP3, pro‐Caspase1, cle‐Caspase1, GSDMD‐FL, and GSDMD‐N) in the renal cortex. Frozen renal cortex tissues were homogenized in RIPA lysis buffer containing protease and phosphatase inhibitors, followed by ice‐cold lysis and centrifugation to collect the supernatant. Protein quantification was performed using the BCA method. Equal amounts of protein samples were subjected to SDS‐PAGE gel electrophoresis, and proteins were then transferred to PVDF membranes. The membranes were blocked with 5% skimmed milk at room temperature for 1 h, followed by incubation with primary antibodies and *β*‐actin (internal Ctrl) at 4°C overnight. On the next day, the membranes were washed with TBST and incubated with corresponding secondary antibodies at room temperature for 1 h. After washing, the membranes were exposed using ECL chemiluminescence detection solution, and band images were captured. ImageJ software was used for densitometric analysis, and the relative protein expression levels were determined by the ratio of the target protein to *β*‐actin.

#### 2.6.4. Immunofluorescence Staining

Mouse kidney tissues were fixed in 4% paraformaldehyde and then embedded in paraffin. Paraffin‐embedded kidney sections (4 *μ*m) were deparaffinized, rehydrated, and fixed. Following antigen retrieval, the sections were blocked with 10% goat serum, and then incubated with the indicated primary antibodies at 4°C overnight. After washing, tissue specimens were incubated with secondary antibodies conjugated with the appropriate fluorophore for 1 h at room temperature. Images were captured using a laser confocal microscope.

#### 2.6.5. RNA Extraction and Quantitative Real‐Time PCR

Mouse kidney tissues were lysed to extract total RNA using TRIzol. PrimeScript II 1st Strand cDNA Synthesis Kit was used to synthesize first‐strand cDNA. RT‐qPCR analysis was performed using SYBR Premix Ex Taq II (Yeasen, China) on a CFX96 Touch Real‐Time PCR Detection System. mRNA levels of target genes were normalized to *Actb*. The 2^−*ΔΔ*CT^ method was performed to calculate the differences in expression levels see Table 1.

#### 2.6.6. In Vitro Mesangial Cell‐Related Indicator Detection

##### 2.6.6.1. Mitochondrial Function‐Related Detection

###### 2.6.6.1.1. Mitochondrial Morphology and Membrane Potential Detection

After treating the mesangial cells, MitoTracker Red and JC‐1 probes were added, and the cells were incubated in the dark according to the reagent kit instructions. Fluorescent images were captured using a laser confocal microscope. ImageJ software was used to quantitatively analyze the average mitochondrial area and branch length and calculate the ratio of red to green fluorescence intensity of JC‐1 to evaluate changes in mitochondrial morphology and membrane potential.

###### 2.6.6.1.2. Cell ATP Content Detection

Cells from each group were collected, and ATP concentration was measured using an ATP assay kit through chemiluminescence. The operation strictly followed the reagent kit instructions, and a standard curve was established using the standard. The ATP content in the samples (nmol/mg) was calculated.

###### 2.6.6.1.3. mtROS Detection

After incubating the cells with mitoSOX Red probe, Hoechst 33342 was added to stain the nuclei. Fluorescence distribution was observed under a laser confocal microscope, and the average signal intensity of mitoSOX red fluorescence was quantitatively analyzed using ImageJ software to reflect the mtROS level.

##### 2.6.6.2. Pyroptosis‐Related Indicator Detection

###### 2.6.6.2.1. Protein Expression Detection

Cells from each group were collected, and total protein was extracted and quantified. Western blotting was used to detect the expression of NLRP3, pro‐Caspase1, cleaved‐Caspase1, GSDMD‐FL, and GSDMD‐N proteins, with *β*‐actin as the internal reference. Gel images were scanned using a gel imaging system, and densitometric analysis was performed with ImageJ software.

###### 2.6.6.2.2. Inflammatory Cytokine and Cell Damage Detection

Cell culture supernatants were collected, and ELISA kits were used to measure the IL‐1*β* concentration. LDH activity was measured using an LDH cytotoxicity detection kit. All operations strictly followed the kit instructions, and absorbance values were read using a microplate reader. IL‐1*β* concentration and LDH activity in the samples were calculated using standard curves.

### 2.7. Statistical Analysis

Data were analyzed using SPSS 21.0 statistical software. Normally distributed quantitative data were presented as mean ± standard deviation (*x* ± *s*). One‐way or two‐way analysis of variance (ANOVA) was used for comparisons among multiple groups, and independent samples *t*‐test was used for pairwise comparisons. A *p* < 0.05 was considered statistically significant.

## 3. Results

### 3.1. AZL Ameliorates Kidney Injury and Inflammation in DKD Model

**Figure 1:**
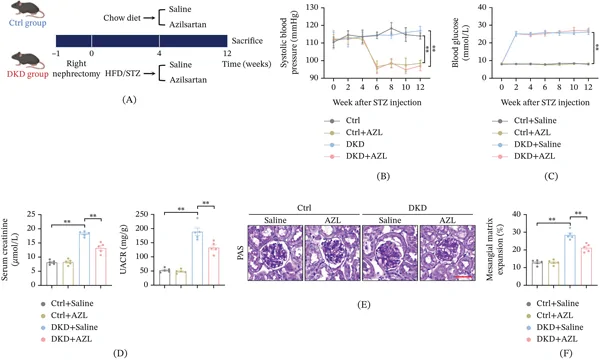
Azilsartan improves renal injury‐related indicators in DKD mice. (A) Schematic diagram of mouse experimental modeling and intervention process. (B) Trend of systolic blood pressure changes in mice from each group. (C) Trend of fasting blood glucose changes in mice from each group. (D) Serum creatinine levels and UACR detection results in mice from each group. (E) PAS‐stained renal tissue sections of mice from each group showing glomerular mesangial matrix. (F) Statistical results of glomerular mesangial matrix expansion rate in renal tissue sections of mice from each group.  ^∗∗^ *p* < 0.01.

Figure 1A shows the experimental animal modeling and intervention process. Compared to the saline group (Ctrl+Saline and DKD+Saline), AZL intervention (Ctrl+AZL and DKD+AZL groups) effectively reduced SBP but had no significant effect on blood glucose (Figure 1B,C). Meanwhile, the serum creatinine and UACR of the DKD+Saline group mice were significantly increased (Figure 1D), and PAS staining of the kidneys showed obvious mesangial matrix expansion (Figure 1E,F), indicating impaired renal function and pathological changes. However, the serum creatinine, UACR levels, and mesangial matrix expansion in the DKD+AZL group were significantly reduced, suggesting that AZL can effectively improve renal function abnormalities and pathological damage in DKD mice.

**Table 1:** PCR primers used in this study.

Gene	Forward primer	Reverse primer
*Actb*	CATTGCTGACAGGATGCAGAAGG	TGCTGGAAGGTGGACAGTGAGG
*Il6*	GAGGATACCACTCCCAACAGACC	AAGTGCATCATCGTTGTTCATACA
*Tnfa*	ATGTCTCAGCCTCTTCTCATTC	GCTTGTCACTCGAATTTTGAGA
*Ccl2*	TCACCTGCTGCTACTCATTCACCA	TACAGCTTCTTTGGGACACCTGCT
*Ccl10*	TGCCACACTCAAAACCAGCAGG	ACACTGCCTGAACGAAGGTCTC

### 3.2. AZL Reduces Inflammatory Response and Pyroptosis Markers in DKD

**Figure 2:**
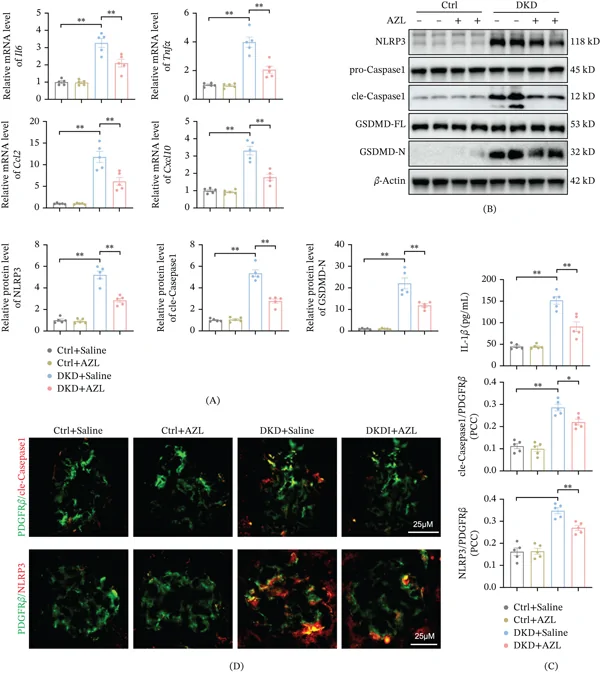
Azilsartan improves kidney tissue inflammation and pyroptosis levels in DKD mice. (A) Relative mRNA expression levels of proinflammatory cytokines IL‐6, TNF‐*α*, CCL2, and CXCL10 in kidney tissue from each group of mice. (B) Western blot images of NLRP3, pro‐Caspase1, cle‐Caspase1, GSDMD‐FL, GSDMD‐N, and the internal reference *β*‐actin in kidney tissue from each group, along with quantitative analysis of protein expression levels of NLRP3, cle‐Caspase1, and GSDMD‐N. (C) IL‐1*β* concentration detection results in serum from each group of mice. (D) Immunofluorescence staining of cle‐Caspase1 and NLRP3 with the marker of mesangial cell (PDGFR*β*). Scale bar, 25 *μ*m.  ^∗∗^ *p* < 0.01.

Compared to the Ctrl groups (Ctrl+Saline and Ctrl+AZL), the DKD+Saline group mice exhibited significantly elevated mRNA expression levels of proinflammatory cytokines Il‐6, TNF‐*α*, CCl2, and CXCl10 in kidney tissue, as well as increased protein expression levels of NLRP3, cleaved‐Caspase1, and GSDMD‐N, along with higher serum IL‐1*β* concentrations (*p* < 0.01). Immunofluorescence staining suggested that NLRP3 and cle‐Caspase1 expression in mesangial cells was increased under DKD conditions, whereas AZL treatment inhibited their expression. These results suggest that the inflammatory response in the kidney tissue and NLRP3 inflammasome‐mediated pyroptosis in DKD mice are significantly enhanced. However, in DKD+AZL group their levels significantly lower than those in the DKD+Saline group (*p* < 0.01). There were no statistically significant differences between Ctrl+Saline and Ctrl+AZL group (*p* > 0.05)(Figure 2A–D).

### 3.3. AZL Alleviates Mitochondrial Dysfunction in DKD

**Figure 3:**
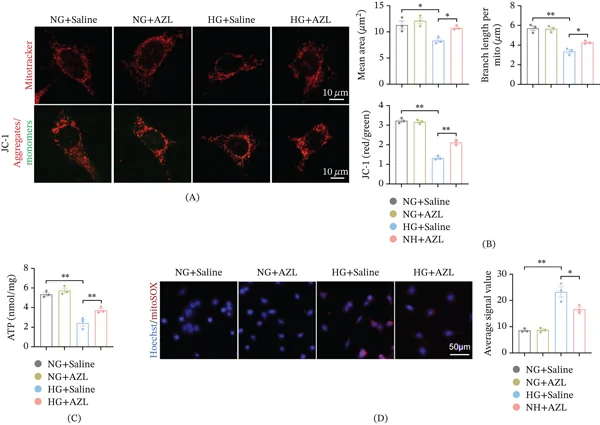
Azilsartan improves mitochondrial function in mesangial cells under high glucose conditions. (A) Representative fluorescent images of mesangial cell mitochondrial morphology (MitoTracker staining, red) and mitochondrial membrane potential (JC‐1 staining, red represents aggregated form, green represents monomeric form) in each group. (B) Quantitative analysis of mitochondrial average area, branch length, and JC‐1 red/green fluorescence ratio in mesangial cells from each group. (C) ATP content detection results in mesangial cells from each group. (D) Representative fluorescent images of mitochondrial reactive oxygen species (mitoSOX, red) and cell nuclei (Hoechst, blue) in mesangial cells from each group, along with quantitative analysis of the average signal intensity.  ^∗^ *p* < 0.05 and  ^∗∗^ *p* < 0.01.

In MMCs, compared to NG+Saline and NG+AZL groups, the HG+Saline group showed significantly decreased mitochondrial average area and branch length (*p* < 0.05), a significant reduction in the JC‐1 red/green fluorescence ratio (*p* < 0.01) (Figure 3B), and a marked decrease in ATP production (*p* < 0.01) (Figure 3C). Additionally, mitoSOX levels were significantly elevated (*p* < 0.01), suggesting that HG induced mitochondrial structural damage, membrane potential decline, metabolic dysfunction, and enhanced oxidative stress in mesangial cells. In the HG+AZL group, the mitochondrial average area, branch length, and JC‐1 red/green ratio significantly improved (*p* < 0.05), ATP production increased (*p* < 0.01), and mitoSOX levels were significantly reduced (*p* < 0.05). No significant differences were observed between NG+Saline and NG+AZL groups (*p* > 0.05) (Figure 3A–D).

### 3.4. AZL Inhibits Mitochondrial ROS and Reduces Cell Damage

**Figure 4:**
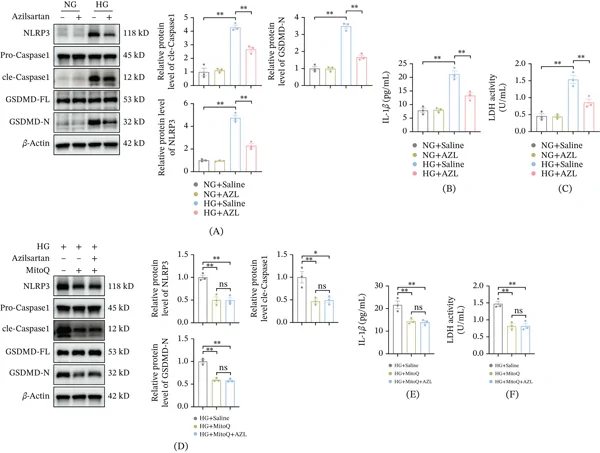
Azilsartan (AZL) improves pyroptosis in mesangial cells under high glucose (HG) conditions by inhibiting mtROS. (A) (left) Western blot analysis and (right) semiquantitative densitometry of pyroptosis‐related proteins including NLRP3, pro‐Caspase1, cle‐Caspase1, GSDMD‐FL, and GSDMD‐N in mesangial cells cultured under normal glucose (NG) or high glucose (HG) and treated with saline or AZL. (B) IL‐1*β* concentration in the culture supernatant of each group. (C) LDH activity in the culture supernatant of each group. (D) Western blot analysis and semiquantitative densitometry of pyroptosis‐related proteins in mesangial cells cultured under HG and treated with saline, the mtROS inhibitor MitoQ, or MitoQ combined with AZL. (E) IL‐1*β* concentration in the culture supernatant of each group. (F) LDH activity in the culture supernatant of each group.  ^∗^ *p* < 0.05 and  ^∗∗^ *p* < 0.01.

Compared to the NG+Saline and NG+AZL groups, the HG+Saline group exhibited significantly increased protein expression levels of NLRP3, cle‐Caspase1, and GSDMD‐N (*p* < 0.01) (Figure 4A,B). Additionally, the concentrations of IL‐1*β* and LDH activity in the supernatant were also significantly elevated (*p* < 0.01) (Figure 4C). In contrast, the HG+AZL group showed a significant reduction in all these indicators (*p* < 0.01) (Figure 4A–C). No statistically significant differences were observed between the NG+Saline and NG+AZL groups for any of the indicators (*p* > 0.05).

### 3.5. AZL Exerts Its Effects Through Inhibition of mtROS

MitoQ, a mitochondrial‐targeted antioxidant, was used to inhibit mtROS generation. In the HG+MitoQ group, the protein expression levels of NLRP3, cle‐Caspase1, GSDMD‐N (Figure 4D), IL‐1*β* concentration (Figure 4E), and LDH activity (Figure 4F) were significantly lower than those in the HG+Saline group (*p* < 0.01). No significant differences were observed between the HG+MitoQ group and the HG+MitoQ+AZL group (*p* > 0.05).

### 3.6. Discussion

In this study, a DKD model was established through unilateral nephrectomy combined with HFD feeding and STZ intraperitoneal injection, which simulated the clinical pathological characteristics of DKD with concomitant renal hemodynamic abnormalities and metabolic disorders, making it more representative of the pathogenesis seen in clinical DKD patients. The renal function and pathological changes observed in this model have a clear disease‐related correlation [9]. The results of this study showed that there were no significant differences in SBP between untreated DKD model mice and normal Ctrl mice. However, AZL intervention reduced SBP in both normal mice and DKD model mice, maintaining stable levels. Notably, this drug had no significant effect on improving hyperglycemia in DKD model mice, confirming that the beneficial effects of angiotensin receptor blockers in improving DKD renal injury are not dependent on glucose‐lowering effects, but rather on blood pressure reduction as an important mechanism [10]. Additionally, DKD model mice exhibited significant renal dysfunction, as evidenced by markedly increased serum creatinine and UACR, as well as typical pathological changes, such as prominent mesangial matrix hyperplasia and expansion in the glomeruli. AZL intervention was able to reverse these renal dysfunction indicators, alleviating pathological damage to the mesangial matrix expansion. Moreover, AZL did not have significant effects on kidney function or pathological structure in normal mice, which fully supports the specificity of angiotensin receptor blockers in improving kidney injury in DKD mice, demonstrating both efficacy and safety in their renal protective effects [11].

The in vivo experimental results of this study show that in the DKD+Saline group, the expression of IL‐1*β*, IL‐6, TNF‐*α*, NLRP3, cle‐Caspase1, and GSDMD‐N in kidney tissue was significantly elevated, which strongly supports the notion that chronic inflammation activation and NLRP3 inflammasome‐mediated pyroptosis are core pathological processes in the development of kidney injury in DKD mice [12]. In the pathological state of DKD, factors such as hyperglycemia can continuously activate local renal inflammation. The massive release of proinflammatory cytokines further induces immune cell infiltration and damage to intrinsic renal cells [13]. The NLRP3 inflammasome, as a key hub in innate immunity, can be abnormally activated to initiate downstream pyroptosis pathways. Through the cleavage of Caspase‐1, GSDMD is cleaved to form GSDMD‐N, which mediates inflammatory programmed cell death in renal tissue while releasing proinflammatory cytokines such as IL‐1*β*. This results in a vicious cycle of “inflammation activation–pyroptosis–inflammation amplification,” which continuously exacerbates mesangial matrix proliferation and renal function damage [14].

This study found that in the DKD+AZL group, the mRNA expression of proinflammatory cytokines, as well as the protein expression of NLRP3 inflammasome and pyroptosis markers in kidney tissue, was significantly suppressed, and the serum IL‐1*β* concentration was significantly lower than in the DKD+Saline group. These findings suggest that AZL effectively ameliorates the chronic inflammation state in the kidneys of DKD mice and blocks NLRP3 inflammasome‐mediated pyroptosis [15]. At the same time, there were no significant differences in the expression of these proinflammatory and pyroptosis‐related indicators between the Ctrl+Saline and Ctrl+AZL groups, which indicates that AZL has no significant effect on kidney inflammation and pyroptosis in normal mice. The regulation of inflammation and pyroptosis by AZL is pathologically specific, exerting its anti‐inflammatory and antipyroptotic effects only under DKD pathological conditions rather than a nonspecific systemic anti‐inflammatory effect. This characteristic is consistent with previous studies in similar organ injury models and drugs [15, 16]. Therefore, AZL targets the inhibition of NLRP3 inflammasome activation, blocking the initiation of downstream pyroptosis pathways from the source. This not only reduces inflammatory cell death in renal tissue but also inhibits the release of proinflammatory cytokines such as IL‐1*β*, successfully breaking the vicious cycle of inflammation and pyroptosis, thus alleviating local renal inflammation and delaying the pathological reconstruction of the glomerulus. This is an important molecular mechanism underlying the renal protective effects of AZL.

This study′s in vitro experiments confirmed that under HG conditions, mesangial cells exhibited mitochondrial fragmentation, significant membrane potential reduction, decreased ATP synthesis, and a substantial increase in mtROS levels, forming a “mitochondrial damage–high mtROS expression–exacerbated damage” vicious cycle. This is the core initiating mechanism of HG‐mediated mesangial cell injury [17, 18]. After AZL intervention, mitochondrial morphological abnormalities induced by HG were significantly reversed, membrane potential stability was restored, ATP generation increased, and mtROS levels were significantly suppressed, repairing mitochondrial damage from both structural and functional aspects. AZL intervention interrupted the vicious cycle of mitochondrial damage and oxidative stress, thereby alleviating oxidative stress damage in mesangial cells. These findings are consistent with previous studies on ARB class drugs [19]. Additionally, under NG conditions, AZL had no significant effect on mesangial cell mitochondrial indicators, which is consistent with the lack of noticeable effects of AZL on normal mouse kidneys in in vivo experiments, further confirming that ARB drugs significantly enhance mitochondrial antioxidant enzyme activity and improve mitochondrial function only in HG/insulin resistance pathological states, with no apparent effect under normal blood glucose conditions. This demonstrates that their mitochondrial protective effects are pathologically specific to hyperglycemia [20]. This study further used MitoQ to specifically inhibit mtROS generation. The results showed that MitoQ significantly reduced HG‐induced pyroptosis and injury‐related indicators in mesangial cells, and its effect was consistent with that of AZL intervention. Moreover, no additive effects were observed with combined MitoQ and AZL intervention, confirming that mtROS is a key upstream signal for activating the NLRP3 pyroptosis pathway under HG conditions [21]. AZL primarily exerts its protective effect on mesangial cells by inhibiting mtROS generation, thereby interrupting the mtROS‐NLRP3‐pyroptosis signaling axis [22].

The renoprotective effects of ARBs in DKD are well established, and the roles of NLRP3/pyroptosis and upstream mtROS in DKD have been described, particularly in tubular cells [23, 24]. Within this mature field, the present study offers three incremental contributions. First, we extend the mtROS–NLRP3–pyroptosis axis to mesangial cells, a cell type central to glomerular pathology but less studied in this context. Second, we demonstrate that AZL—a high‐affinity ARB—suppresses this axis in mesangial cells, distinguishing its mechanism from other ARBs (e.g., irbesartan) whose NLRP3 inhibition was linked to Nrf2 rather than direct mtROS modulation [25]. Third, using MitoQ as an independent mtROS scavenger, we provide causal evidence that mtROS lies upstream of NLRP3 activation in AZL′s action, moving beyond correlative observations. Collectively, these findings refine our understanding of AZL′s renoprotective profile in DKD.

Several limitations of the present study should be acknowledged. Firstly, although AZL significantly lowered blood pressure in DKD mice, we did not include a non‐ARB antihypertensive comparator (e.g., a calcium channel blocker) titrated to achieve equivalent blood pressure reduction. Therefore, the relative contributions of hemodynamic versus nonhemodynamic mechanisms to the observed renoprotection cannot be precisely determined. Secondly, although we demonstrated that AZL inhibits mtROS generation and NLRP3‐mediated pyroptosis in cultured mesangial cells, we did not measure mtROS levels in vivo. Consequently, the in vivo relevance of the mtROS/pyroptosis pathway remains inferential. Future studies employing intravital imaging of mitochondrial ROS, or conditional knockout models targeting the NLRP3 pathway specifically in mesangial cells, would help establish causality. Another limitation is that we did not perform transmission electron microscopy (TEM) to directly visualize mitochondrial ultrastructure in renal tissues. Future studies incorporating TEM evaluation will be necessary to provide definitive ultrastructural confirmation of the mitochondrial protective effects of AZL in DKD. Additionally, in our in vitro experiments, HG was compared with NG without an equimolar mannitol osmotic Ctrl.

In conclusion, this study demonstrates that AZL effectively improves kidney injury in DKD mice and attenuates HG‐induced mesangial cell damage. In vitro experiments reveal that AZL inhibits mtROS overproduction, thereby blocking NLRP3 inflammasome activation, caspase‐1 cleavage, GSDMD‐N formation, and subsequent pyroptosis in mesangial cells.

## Author Contributions

W.L. conceived and designed the research and wrote the original draft of the manuscript. F.H. conducted the experiments and draw the figures. X.L. curated and analyzed the data.

## Funding

This work was supported by the 2025 Hangzhou Medical and Health Science and Technology Project, B20261672.

## Disclosure

The study procedure followed all relevant standards, regulations, and laws. All authors have read and approved the final version of the manuscript. Corresponding author had full access to all of the data in this study and takes complete responsibility for the integrity of the data and the accuracy of the data analysis.

## Ethics Statement

All procedures complied with ARRIVE guidelines (see File S1: ARRIVE guidelines 2.0 author checklist). All the experiments were approved by the Animal Ethics Committee of The Second Affiliated Hospital of Zhejiang University School of Medicine (Ethical Approval No.: AIRB‐2026‐2090). All efforts were made to minimize the number of animals used and to reduce their suffering throughout the experimental process. No human subjects were involved in this study, so consent to participate was not applicable.

## Consent

The authors have nothing to report.

## Conflicts of Interest

The authors declare no conflicts of interest.

## Supporting information


**Supporting Information** Additional supporting information can be found online in the Supporting Information section. File S1: ARRIVE guidelines 2.0 author checklist (PDF). This file contains the completed ARRIVE Essential 10 checklist for this study.

## Data Availability

Data sharing not applicable to this article as no datasets were generated or analysed during the current study.

## References

[1] Gupta S. , Dominguez M. , and Golestaneh L. , Diabetic Kidney Disease: An Update, Medical Clinics of North America. (2023) 107, no. 4, 689–705, 10.1016/j.mcna.2023.03.004. 37258007

[2] Dwivedi S. and Sikarwar M. S. , Diabetic Nephropathy: Pathogenesis, Mechanisms, and Therapeutic Strategies, Hormone and Metabolic Research. (2025) 57, no. 1, 7–17, 10.1055/a-2435-8264, 39572154. 39572154

[3] Mima A. , Matsubara T. , Arai H. , Abe H. , Nagai K. , Kanamori H. , Sumi E. , Takahashi T. , Iehara N. , Fukatsu A. , Kita T. , and Doi T. , Angiotensin II-Dependent Src and Smad 1 Signaling Pathway Is Crucial for the Development of Diabetic Nephropathy, Laboratory Investigation. (2006) 86, no. 9, 927–939, 10.1038/labinvest.3700445, 16767106. 16767106

[4] Mima A. , Arai H. , Matsubara T. , Abe H. , Nagai K. , Tamura Y. , Torikoshi K. , Araki M. , Kanamori H. , Takahashi T. , Tominaga T. , Matsuura M. , Iehara N. , Fukatsu A. , Kita T. , and Doi T. , Urinary Smad 1 Is a Novel Marker to Predict Later Onset of Mesangial Matrix Expansion in Diabetic Nephropathy, Diabetes. (2008) 57, no. 6, 1712–1722, 10.2337/db07-1726, 18285555. 18285555

[5] Al Mamun A. , Ara Mimi A. , Wu Y. , Zaeem M. , Aziz M. A. , Suchi S. A. , Alyafeai E. , Munir F. , and Xiao J. , Pyroptosis in Diabetic Nephropathy, Clinica Chimica Acta. (2021) 523, 131–143, 10.1016/j.cca.2021.09.003. 34529985

[6] Chen Y. , Chen R. , Ji X. , Zeng Z. , and Guan C. , NLRP3 Inflammasome-Mediated Pyroptosis in Diabetic Nephropathy: Pathogenic Mechanisms and Therapeutic Targets, Journal of Inflammation Research. (2025) 18, 8399–8418, 10.2147/JIR.S524246, 40585041. 40585041 PMC12206412

[7] Khan Q. A. , Sharma S. , Mulk I. U. , Li D. , Belay N. F. , Afzal M. , Farrukh A. M. , Asad M. , Baqi A. , and Semakieh B. , Effect of Azilsartan on Clinical Blood Pressure Reduction Compared to Other Angiotensin Receptor Blockers: A Systematic Review and Meta-Analysis, Annals of Medicine and Surgery. (2024) 86, no. 2, 958–967, 10.1097/MS9.0000000000001547, 38333313. 38333313 PMC10849446

[8] Hou Y. , Tan E. , Shi H. , Ren X. , Wan X. , Wu W. , Chen Y. , Niu H. , Zhu G. , Li J. , Li Y. , and Wang L. , Mitochondrial Oxidative Damage Reprograms Lipid Metabolism of Renal Tubular Epithelial Cells in the Diabetic Kidney, Cellular and Molecular Life Sciences. (2024) 81, no. 1, 10.1007/s00018-023-05078-y, 38200266. PMC10781825 38200266

[9] Lan T. , Tang T. , Li Y. , Duan Y. , Yuan Q. , Liu W. , Ren Y. , Li N. , Liu X. , Zhang Y. , Li X. , Jin G. , Wang S. , and Guo J. , FTZ Polysaccharides Ameliorate Kidney Injury in Diabetic Mice by Regulating Gut-Kidney Axis, Phytomedicine. (2023) 118, 154935, 10.1016/j.phymed.2023.154935, 37364420. 37364420

[10] Angeloni E. , Azilsartan Medoxomil in the Management of Hypertension: An Evidence-Based Review of Its Place in Therapy, Core Evidence. (2016) 11, 1–10, 10.2147/CE.S81776, 27103882. 27103882 PMC4829189

[11] Mohany M. , Ahmed M. M. , and Al-Rejaie S. S. , The Role of NF-*κ*B and Bax/Bcl-2/Caspase-3 Signaling Pathways in the Protective Effects of Sacubitril/Valsartan (Entresto) Against HFD/STZ-Induced Diabetic Kidney Disease, Biomedicines. (2022) 10, no. 11, 10.3390/biomedicines10112863, 36359384. PMC9717728 36359384

[12] Xiao S. Y. , Lv Y. H. , Ji Y. M. , Dong Y. , Liu M. C. , Li T. , Cui X. R. , and Hu Y. , The NLRP3 Inflammasome: A Pivotal Orchestrator of Multisystem Diseases-From Molecular Mechanisms to Therapeutic Innovation, Molecular Biology Reports. (2025) 52, no. 1, 10.1007/s11033-025-11116-8, 41085563. PMC12521272 41085563

[13] Youssef N. , Noureldein M. H. , Riachi M. E. , Haddad A. , and Eid A. A. , Macrophage Polarization and Signaling in Diabetic Kidney Disease: A Catalyst for Disease Progression, American Journal of Physiology-Renal Physiology. (2024) 326, no. 3, F301–F312, 10.1152/ajprenal.00266.2023, 38153850. 38153850

[14] Fan Q. , Li R. , Wei H. , Xue W. , Li X. , Xia Z. , Zhao L. , Qiu Y. , and Cui D. , Research Progress of Pyroptosis in Diabetic Kidney Disease, International Journal of Molecular Sciences. (2024) 25, no. 13, 10.3390/ijms25137130, 39000237. PMC11241146 39000237

[15] Pan Y. , Liu L. , Yang H. , Chen W. , Chen Z. , and Xu J. , Sacubitril/Valsartan Improves Progression of Early Diabetic Nephropathy in Rats Through Inhibition of NLRP3 Inflammasome Pathway, Diabetes, Metabolic Syndrome and Obesity. (2022) 15, 2479–2488, 10.2147/DMSO.S366518, 35992034. PMC9386175 35992034

[16] Raheem N. M. and Mohammed Ali Mahmood N. , Azilsartan Suppresses the Antiapoptotic Biomarker and Pro-Inflammatory Cytokines in Rat Model of Cisplatin-Induced Retinal and Optic Nerve Toxicity, Human & Experimental Toxicology. (2023) 42, 9603271231155092, 10.1177/09603271231155092, 36930951. 36930951

[17] Sun C. , Li Y. , Tao Y. , Wu Q. , Liu B. , and Zhou Y. , Imbalance of Mitochondria and Abnormalities in Lipid Metabolism in Diabetic Tubulopathy, Annals of Medicine. (2026) 58, no. 1, 2615478, 10.1080/07853890.2026.2615478, 41550024. 41550024 PMC12818318

[18] Mima A. , Mitochondria-Targeted Drugs for Diabetic Kidney Disease, Heliyon. (2022) 8, no. 2, e08878, 10.1016/j.heliyon.2022.e08878, 35265754. 35265754 PMC8899696

[19] Zhang X. J. , Liu C. C. , Li Z. L. , Ding L. , Zhou Y. , Zhang D. J. , Zhang Y. , Hou S. T. , and Ma R. X. , Sacubitril/Valsartan Ameliorates Tubulointerstitial Fibrosis by Restoring Mitochondrial Homeostasis in Diabetic Kidney Disease, Diabetology & Metabolic Syndrome. (2024) 16, no. 1, 10.1186/s13098-024-01284-1, 38341600. PMC10858505 38341600

[20] Godoy-Lugo J. A. , Thorwald M. A. , Mendez D. A. , Rodriguez R. , Nakano D. , Nishiyama A. , and Ortiz R. M. , Glucose Increases Hepatic Mitochondrial Antioxidant Enzyme Activities in Insulin Resistant Rats Following Chronic Angiotensin Receptor Blockade, International Journal of Molecular Sciences. (2022) 23, no. 18, 10897, 10.3390/ijms231810897, 36142809. 36142809 PMC9505141

[21] Rimessi A. , Previati M. , Nigro F. , Wieckowski M. R. , and Pinton P. , Mitochondrial Reactive Oxygen Species and Inflammation: Molecular Mechanisms, Diseases and Promising Therapies, International Journal of Biochemistry & Cell Biology. (2016) 81, Part B, 281–293, 10.1016/j.biocel.2016.06.015. 27373679

[22] Liu H. , Mao P. , Wang J. , Wang T. , and Xie C. H. , Azilsartan, an Angiotensin II Type 1 Receptor Blocker, Attenuates Tert-Butyl Hydroperoxide-Induced Endothelial Cell Injury Through Inhibition of Mitochondrial Dysfunction and Anti-Inflammatory Activity, Neurochemistry International. (2016) 94, 48–56, 10.1016/j.neuint.2016.02.005, 26879328. 26879328

[23] Wu J. , Sun Z. , Yang S. , Fu J. , Fan Y. , Wang N. , Hu J. , Ma L. , Peng C. , Wang Z. , Lee K. , He J. C. , and Li Q. , Kidney Single-Cell Transcriptome Profile Reveals Distinct Response of Proximal Tubule Cells to SGLT2i and ARB Treatment in Diabetic Mice, Molecular Therapy. (2022) 30, no. 4, 1741–1753, 10.1016/j.ymthe.2021.10.013, 34678510. 34678510 PMC9077318

[24] Lv J. , Perkovic V. , Foote C. V. , Craig M. E. , Craig J. C. , Strippoli G. F. , and Cochrane Kidney and Transplant Group , Antihypertensive Agents for Preventing Diabetic Kidney Disease, Cochrane Database of Systematic Reviews. (2013) 2013, no. 8, CD004136, 10.1002/14651858.CD004136.pub3. PMC11357690 23235603

[25] Li Y. , Long W. , Zhang H. , Zhao M. , Gao M. , Guo W. , and Yu L. , Irbesartan Ameliorates Diabetic Nephropathy by Activating the Nrf 2/Keap 1 Pathway and Suppressing NLRP3 Inflammasomes In Vivo and In Vitro, International Immunopharmacology. (2024) 131, no. 131, 111844, 10.1016/j.intimp.2024.111844. 38503013

